# Supplementary material for: Evaluation of Everolimus Activity against Mycobacterium tuberculosis Using In Vitro Models of Infection
Source: Antibiotics (Basel). 2023 Jan 13;12(1):171. doi: 10.3390/antibiotics12010171 (PMC9854797; doi:10.3390/antibiotics12010171)
Supplement: Supplementary file 1 [file antibiotics-12-00171-s001.zip › antibiotics-2146023-supplementary.pdf]

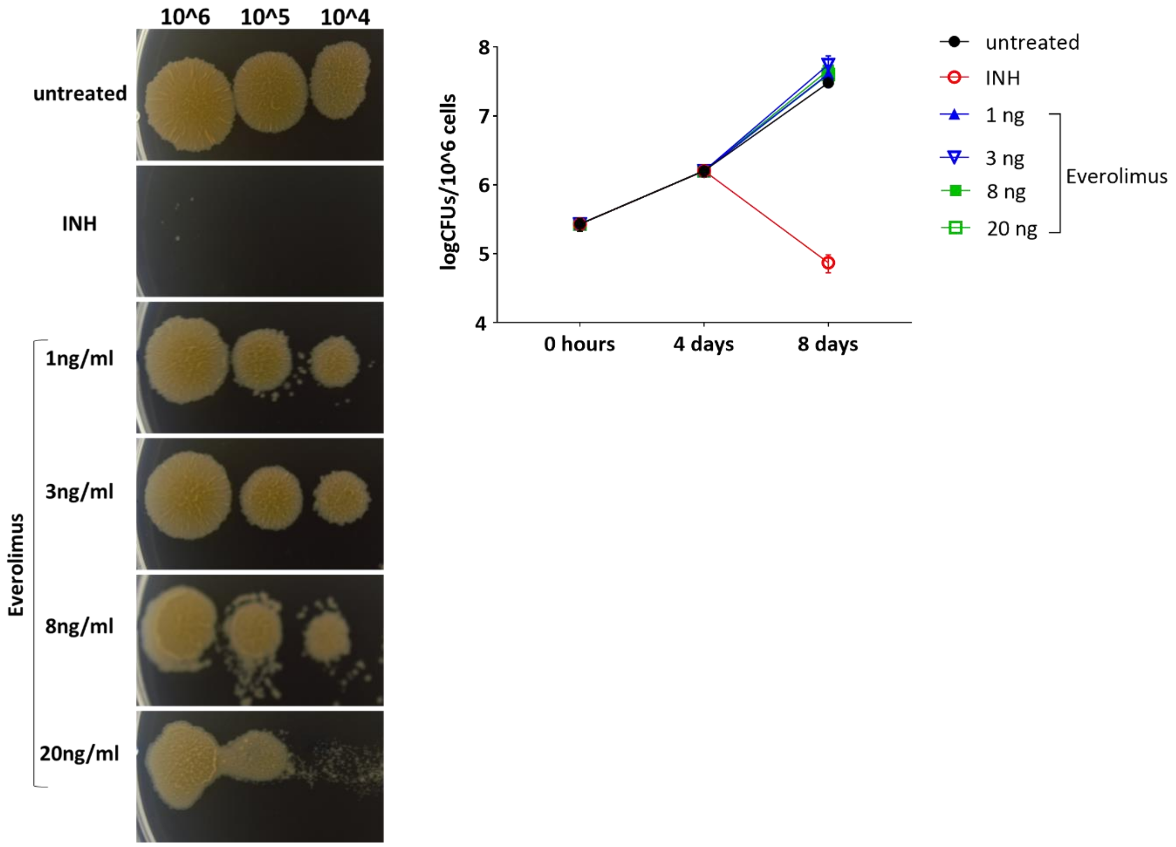

Supplementary Figure 1. Growth rate of the mycobacterial strains used for the infection modes. Mycobacterial growth was evaluated plating *Mtb* H37Rv (ranged from 10^6 CFUs/ml to 10^4 CFUs/ml) on 7H11 solid medium containing Everolimus at final concentrations of 1ng/ml, 3ng/ml, 8ng/ml and 20ng/m or INH (0.2µg/ml). The same Everolimus concentrations were used to assess its immunomodulating activity on ex vivo PBMCs infection model. H37Rv was used to infect peripheral blood mononuclear cells (PBMCs) at MOI = 1:10 with respect to monocytes. Everolimus (1ng/mL, 3ng/m, 8ng/mL and 20ng/mL) and INH (0.2ug/mL) were added four-day post infection. Colony forming units (CFUs) analysis was performed 8-days post infection. CFUs are reported as average ± SD in log10 scale.
